# High Permittivity Nanocomposites Embedded with Ag/TiO_2_ Core–Shell Nanoparticles Modified by Phosphonic Acid

**DOI:** 10.3390/polym10060586

**Published:** 2018-05-27

**Authors:** Xizi Chen, Fei Liang, Wenzhong Lu, Zheng Jin, Yifei Zhao, Ming Fu

**Affiliations:** 1 School of Optical and Electronic Information, Huazhong University of Science and Technology, Wuhan 430074, China; 15671677452@163.com (X.C.); lwz@mail.hust.edu.cn (W.L.); m201772240@hust.edu.cn (Z.J.); u201313965@mail.hust.edu.cn (Y.Z.); fuming@hust.edu.cn (M.F.); 2 Key Lab of Functional Materials for Electronic Information (B), Ministry of Education, Wuhan 430074, China

**Keywords:** core–shell structure, interfacial modification, dielectric properties, nanocomposites

## Abstract

In this paper, nanocomposites that contain core-shell Ag/TiO_2_ particles as the filler and polytetrafluoroethylene (PTFE) as the matrix were investigated. Two surfactants, namely octyl phosphonic acid (OPA) and pentafluorobenzyl phosphonic acid (PFBPA), were applied to modify Ag/TiO_2_ fillers for uniform dispersion in the matrix. Fourier transform infrared spectroscopy analysis of bonds between the TiO_2_ shells and the phosphonic modifiers shows Ti–O–P chemical bonding between the Ag/TiO_2_ fillers and the modifiers. Thermogravimetric analysis results show a superior adsorption effect of PFBPA over OPA on the Ag/TiO_2_ filler surface at the same weight percentage. For nanocomposites that contain modified Ag/TiO_2_ nanoparticles, the loss was reduced despite the high permittivity at the same loading. The permittivity of the nanocomposites by PFBPA is larger than that of OPA, because the more uniform dispersion of inorganic particles in the PTFE matrix enhances the interfacial polarization effect. The mechanism of enhanced dielectric performance was studied and discussed.

## 1. Introduction

Polymer-based composites have been intensively studied for their applications in electronic and microwave devices [1,2,3,4]. Polymers have a number of attractive properties, such as low processing temperature, low leakage current, and low Young’s modulus at relatively low permittivity and thermal conductivity. To meet the developing trends of miniaturization and integration of electronic systems, various different fillers have been embedded in polymers [5]. Among the various structures for fillers, a filler with a core-shell structure has attracted considerable interest, owing to its combination of favorable core and shell properties [6,7,8].

**Figure 1:**
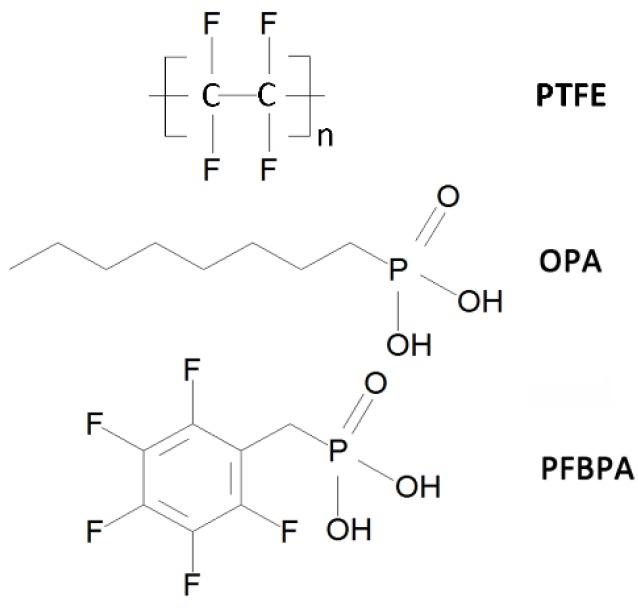
Molecular structures of polytetrafluoroethylene (PTFE), octyl phosphonic acid (OPA), and pentafluorobenzyl phosphonic acid (PFBPA).

Silver nanoparticles (NPs) were chosen as the core in our study because of their excellent thermal, electric, and optical performance. Much research has focused on finding a suitable insulator/semiconductor shell for silver NPs, to reduce the loss of silver-polymer composites [9,10]. Organic shells are compatible with the polymer matrix to ensure the dispersion of fillers in the matrix without coupling agents. Dang et al. prepared nanocomposites with a PVDF matrix embedded with Ag/polydopamine core–shell NPs, and achieved the highest dielectric constant (*ε_r_* ≈ 53) at a relatively low dielectric loss (0.52) [11]. Nan and coworkers synthesized Ag/C core–shell hybrid particles, and the organic nanoshells functioned as electrical barriers between the Ag cores, to form a continuous interparticle barrier layer network [12]. Nevertheless, the low value *ε_r_* and the poor thermostability of the organic material confines the *ε_r_* of the whole core-shell particles, and restricts the sintering temperature of the polymer-based composites, respectively. Inorganic materials such as TiO_2_ [13] and SiO_2_ [14] have functioned as shells in many works. The P(VDF-HFP)-based composites that contain Ag/TiO_2_ core-shell NPs, and the hybrid films fabricated by Zhang et al., exhibited enhanced *ε_r_* near 40 at 10 vol % filler loading [15]. Wang et al. reported Ag/SiO_2_/PI nanocomposites with a maximum thermal conductivity of 7.88 W/(mK) and relative permittivity and dielectric loss of approximately 11.7 and 0.015 at 1 MHz, respectively [14]. Rutile TiO_2_ was chosen as an insulating shell in our study. This is firstly due to is its high dielectric constant and good electrical properties. Secondly, the TiO_2_ shells cut off the conductive path through Ag nanoparticles, thereby suppressing the leakage current of the resulting nanocomposites. However, polymer and inorganic shells have different surface energies. When inorganic core-shell fillers directly embed into the organic polymer, poor compatibility between the two leads to inhomogeneity or cracks in composites, especially at a high volume fraction (≥50 vol %). Coupling agents, with hydrophilic groups at one end and lipophilic groups at the other, improve the wetting between hydrophilic fillers and lipophilic polymer by producing chemical bonds [16,17,18]. Ehrhardt and coworkers employed pentafluorobenzyl phosphonic acid (PFBPA) to modified CaCu_3_Ti_4_O_12_, and obtained an *ε_r_* value enhanced by a factor of 5 compared with the pure P(VDF-HFP), despite the fact that their loss tangents remained low (<0.1) [19]. Perry et al. reported that BaTiO_3_ particles modified by suitable phosphonic acids disperse well in polycarbonate [20]. Phosphonic acids can also modify TiO_2_ by readily forming stable Ti–O–P bonds [21,22]. For Ag/TiO_2_ core-shell NPs, one major challenge in our work was to choose suitable modifiers for fillers that disperse uniformly in the PTFE matrix, thus raising the overall quality of nanocomposites. By applying phosphonic acids to keep the Ti–O–P bonds stable and to improve the lipophilicity of Ag/TiO_2_ core–shell NPs, we investigated the dielectric property of modified Ag/TiO_2_/PTFE nanocomposites. We choose PTFE as the polymer matrix because of its low dielectric loss and relatively stable dielectric properties at high frequencies. The chemical structure of PTFE is shown in Figure 1.

In this paper, we develop a facile and general method for the synthesis of a PTFE-based nanocomposite that contains surface functionalized Ag/TiO_2_ core-shell NPs [23,24,25]. We used nano-silver and tetrabutyl titanate as raw materials, and acetylacetonate and acetic acid as hydrolysis inhibitors, to successfully prepare Ag/TiO_2_ NPs. Two phosphonic acids were used as modifiers to adjust the lipophilicity of the Ag/TiO_2_ NPs. The structures of the OPA and PFBPA are shown in Figure 1. After modification by the two surfactants, the Ag/TiO_2_ NPs were characterized by Fourier transform infrared spectroscopy (FTIR) and thermogravimetric analysis (TGA). The FTIR and TGA results show that surface modifiers form robust chemical bonds (Ti–O–P) with Ag/TiO_2_ NPs. On this basis, the dielectric properties and conductivities of the PTFE polymer embedded by modified Ag/TiO_2_ NPs were measured. The use of phosphonic acids leads to a more homogeneous particle distribution, and improves the dielectric properties of the whole composite. Theoretical models, including percolation theory (PT) [26], effective medium theory (EMT) [27], and effective medium percolation theory (EMPT) [28], were compared with the experimental *ε_r_* of the Ag/TiO_2_/PTFE nanocomposites.

## 2. Materials and Methods

All chemicals were obtained from National Chemicals Reagent Co., Ltd. unless stated otherwise. Ag/TiO_2_ core–shell NPs were synthesized by the sol-gel method. Ag NPs (Aladdin) were dissolved in ethanol and dispersed evenly by ultrasonication. This solution was mixed with a solution that contained tetrabutyl titanate (99.0%, Aladdin), acetylacetonate, acetic acid, and ethanol, under constant stirring. Then, deionized water was added dropwise to the prepared solution to trigger the slow hydrolysis of tetrabutyl titanate, and induce the formation of shells around the metal core. A dark brown gel was formed after stirring for 1 h at 65 °C, and then dried at 80 °C, developing green-black amorphous Ag/TiO_2_ powder. After calcination at 800 °C for 2 h, rutile Ag/TiO_2_ NPs were prepared. The calcinated particles were finely pulverized with deionized water by using a planetary ball mill for 2 h.

Ag/TiO_2_ core–shell NPs were ultrasonically dispersed in ethanol. Surface modifiers were added to this dispersion while stirring, and the weight ratio of modifiers and Ag/TiO_2_ power was 2 wt %. Then, the mixed liquor was handled by extensive ethanol washing and centrifugation to remove excess free surfactant molecules. The precipitate was dried to obtain the modified Ag/TiO_2_ powder.

The PTFE-based composites were prepared by a solution blended process. The PTFE emulsion was supplied by China Zhonghao Chenguang Research Institute Co. Ltd. The modified Ag/TiO_2_ powder was weighed in stoichiometric ratio and mixed into PTFE emulsions at varying volumetric proportions (40, 50, 60, and 70 vol %). After 30 min of ultrasonic dispersion and 2 h of stirring, the solution was dried at 80 °C for 12 h. The dry mixture was obtained and pulverized. The powder was molded to tablet samples with a diameter of approximately 10 mm and a thickness of 1 mm at 15 MPa. The samples were calcined at 370 °C for 2 h, yielding the nanocomposite sample.

The sample structure was investigated by X-ray diffraction (XRD-7000, Shimadzu, Kyoto, Japan) with Cu Kα1 radiation (λ = 0.154056 nm) over the range 20° ≤ 2*θ* ≤ 80°. The size distribution of Ag/TiO_2_ core-shell NPs were measured by a laser particle analyzer (Mastersizer 3000, Malvern Panalytical, Malvern, UK). Field emission scanning electron microscopy (SEM; JSM-7600F, JEOL, Tokyo, Japan) and transmission electron microscopy (TEM; JEM-1230, JEOL, Tokyo, Japan) were used to observe the dispersion of NPs in the composites and the Ag/TiO_2_ core–shell structure. FTIR spectra were obtained on a Bruker TENSOR spectrometer (Bruker, Karlsruhe, Germany) at a resolution of 0.5 cm^−1^. TGA was conducted using Diamond TG at a heating rate of 10 °C/min. The dielectric properties and conductivity of the composite were measured with an impedance analyzer (Agilent 4294A, Agilent Technologies, Santa Clara, CA, USA) at room temperature.

## 3. Results and Conclusions

### 3.1. Characterization of Surfactant-Coated Ag/TiO_2_ Core–Shell NPs

**Figure 2:**
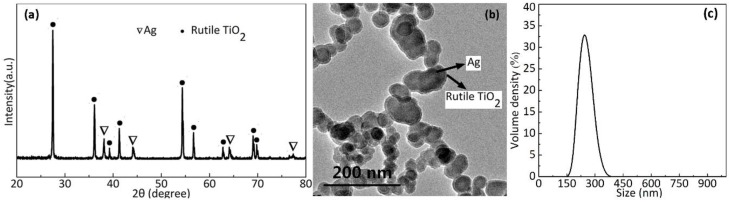
(**a**) X-ray diffraction (XRD) patterns from Ag/TiO_2_ core-shell nanoparticles (NPs) with different surfactants, (**b**) TEM image of Ag/TiO_2_ core-shell NPs, and (**c**) the size distribution of Ag/TiO_2_ core–shell NPs.

Figure 2a shows the XRD patterns from Ag/TiO_2_ core–shell samples, indicating perfect lines that correspond to Ag and rutile TiO_2_. The 2θ values at 38.1°, 44.2°, 64.2°, and 77.6° correspond to (111), (200), (220), and (311) crystal planes of metallic silver, respectively. Similarly, the nine remaining distinct peaks of TiO_2_ are obtained. The diffraction peak of AgO in the XRD spectrum is undetectable, thereby proving that the TiO_2_ shells on the surface of Ag cores prevent the oxidation of metallic silver. The XRD results correspond with the literature [23,24]. Figure 2b shows the morphology of the Ag/TiO_2_ core-shell NPs observed by transmission electron microscopy (TEM), where Ag cores represent the dark gray core area that ranges from 40 nm to 90 nm, while the TiO_2_ shells represent the gray sheath with an area of approximately 8 nm to 10 nm. The TEM image indicates the uniform coating of ellipsoidal silver cores with TiO_2_. Figure 2c shows the size distribution of Ag/TiO_2_ core-shell NPs in ethanol. The size of most agglomeration containing several Ag/TiO_2_ NPs is approximately 220 nm.

**Figure 3:**
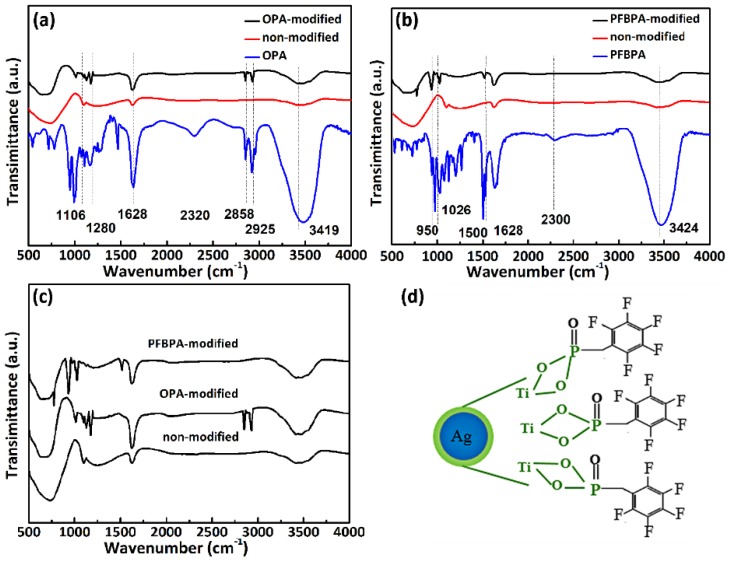
FTIR spectra of Ag/TiO_2_ NPs treated with different surfactants: (**a**) OPA; (**b**) PFBPA; (**c**) comparison diagram of OPA-modified, PFBPA-modified, and non-modified; (**d**) proposed surface structure of Ag/TiO_2_ NPs modified with PFBPA.

On the basis of the synthesis of core-shell NPs, two surfactants were applied to modify the surface of Ag/TiO_2_ NPs, and improve the wetting between NPs and the organic host material. After triple ethanol washing, Ag/TiO_2_ NPs were characterized by FTIR spectroscopy. Figure 3a–c shows the FTIR spectra of the Ag/TiO_2_ NPs, surfactants and the modified Ag/TiO_2_ NPs, respectively. According to Figure 3a, the –CH_3_ and –CH_2_ stretching modes (2925 and 2858 cm^−1^) were detected in modified NPs, but not in non-modified NPs, thereby proving the OPA binding on Ag/TiO_2_ NPs. Major changes in the number and frequencies of the P=O and P–O stretching bands (between 1106 and 1280 cm^−1^ for OPA-modified) in Figure 3a indicate the tridentate surface bonding of OPA on TiO_2_ shells [20]. Meanwhile, the disappearance of P–O–H stretching bands (2320 cm^−1^ for OPA) molecules and the inorganic shells are consistent with previous studies of phosphonic acids bound to TiO_2_ [20,29]. As shown in Figure 3b, a similar phenomenon appears in the PFBPA-modified powder. The structure of a PFBPA-modified Ag/TiO_2_ nanoparticle is schematically shown in Figure 3d, where the blue area, the green boundary, and the black region represent the Ag core, the TiO_2_ shell, and coupling molecules, respectively. Apart from the change of P=O and P–O stretching bands in PFBPA-modified NPs, the characteristic absorbance of PFBPA at 1500 cm^−1^, aromatic vibration, and the C–F stretching peaks were detected in PFBPA-modified samples. In contrast, the characteristic peaks of the surfactant were undetectable in non-modified samples. The FTIR results show that the two surfactants form a stable bond with titanium dioxide shells.

**Figure 4:**
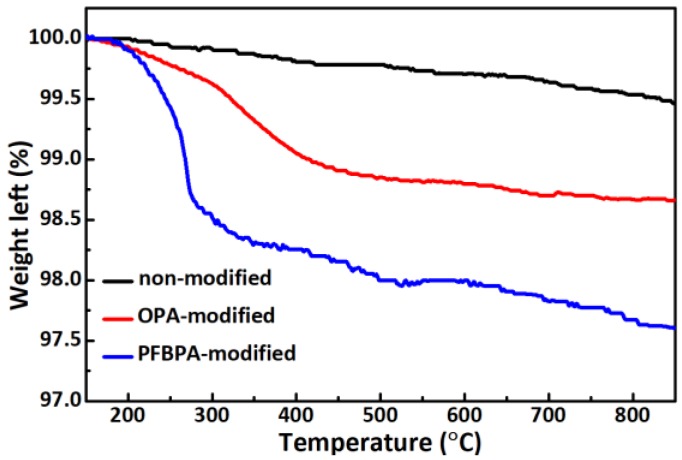
Thermogravimetric analysis of non-modified, OPA-modified, and PFBPA-modified Ag/TiO_2_ powders.

The amount of surface-bonded coupling molecules in non-modified and modified Ag/TiO_2_ NPs was quantified by TGA, as shown in Figure 4. The weight ratio of modifiers and Ag/TiO_2_ power is initially 2 wt %. For non-modified samples, the weight loss (~0.3% at 850 °C) is only minimal, possibly as a result of residual organics from the preparation process. By contrast, significant weight loss was observed for modified Ag/TiO_2_ samples, thereby indicating the thermal decomposition of phosphonic coupling agents. PFBPA-modified powders obtained a higher weight decrease than OPA-modified powders. The weight loss corresponds to a surface coverage of ligands [19]. When heating reached 850 °C, the relative weight left of OPA- and PFPBA-modified powders were 0.79% and 1.86%, respectively, thereby indicating the comparatively high and robust surface coverage of Ag/TiO_2_ NPs by the PFBPA modifier.

### 3.2. Morphology and Dielectric Properties of Modified Ag/TiO_2_/PTFE Nanocomposite

**Figure 5:**
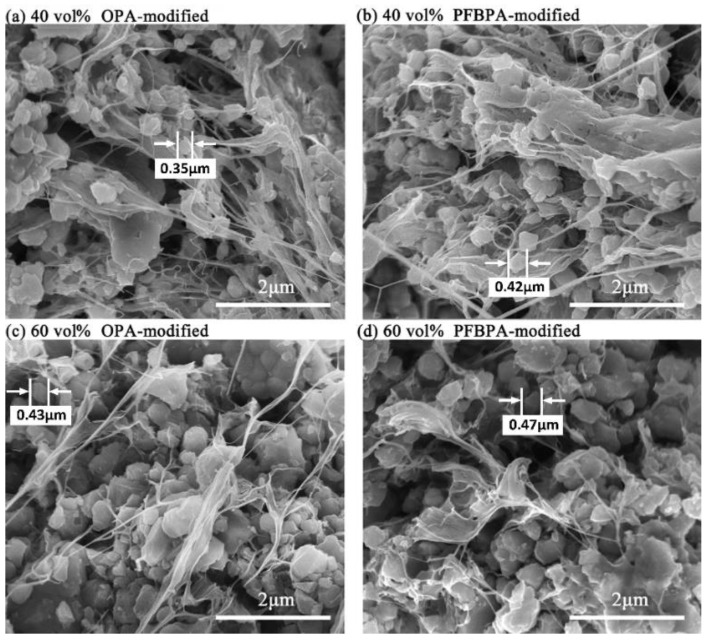
SEM images of freshly fractured cross-sections of Ag/TiO_2_/PTFE nanocomposites containing modified Ag/TiO_2_ in loadings of 40 vol % and 60 vol %. (**a**,**c**): OPA-modified NPs, (**b**,**d**): PFBPA-modified NPs.

SEM images of freshly fractured cross-sections of Ag/TiO_2_/PTFE nanocomposites containing modified Ag/TiO_2_ at 40 and 60 vol % loading are depicted in Figure 5. Ag/TiO_2_ core-shell NPs appear as white dots, and Ag NPs are coated by TiO_2_ shells. PTFE appear as dendritic crystals and other amorphous vitreous body. Compared with the composites at 40 vol % loading, the continuity of the polymer chain at 60 vol % loading degrades, possibly because of nanoparticle agglomeration in the matrix [29]. The SEM images in Figure 5a,b show an increased degree of dispersion, thereby illustrating the graded functionalized effect by the two modifiers. Compared with OPA-modified NPs, PFBPA-modified NPs are more uniformly dispersed throughout the PTFE matrix, thereby demonstrating good compatibility with PTFE achieved by PFBPA. This outcome basically agrees with the TGA results, thus proving that PFBPA forms robust chemical bonds with the Ag/TiO_2_ NPs, for stronger lipophilicity.

**Figure 6:**
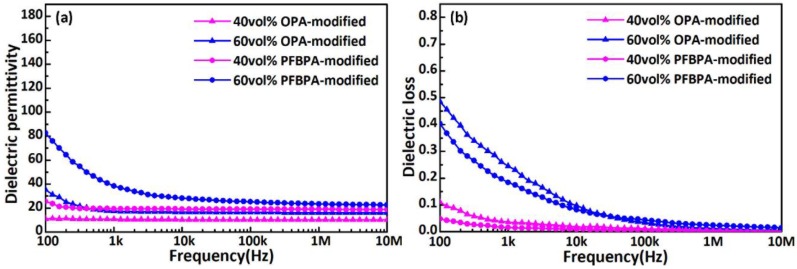
Frequency dependence of (**a**) dielectric permittivity and (**b**) dielectric loss for nanocomposites at 40 vol % and 60 vol % nanoparticles loading with OPA-modified and PFBPA-modified Ag/TiO_2_ filler at room temperature.

Figure 6 shows the frequency dependence of dielectric performance for Ag/TiO_2_/PTFE nanocomposites at room temperature when the Ag/TiO_2_ NPs undergo modification by OPA and PFBPA. According to Figure 6, dielectric permittivity and loss below 10 kHz decrease when the frequency increases during the relaxation of the interface polarization [30]. As the frequency increases to 10 kHz–10 MHz, electronic displacement polarization becomes important in various polarizations. Therefore, *ε_r_* and loss at 10 kHz–10 MHz remain low and become frequency-independent. The dielectric permittivity and loss have a significant dependence on volume fraction and modifiers. Modifiers degrade the dispersion of modified Ag/TiO_2_ NPs in the PTFE matrix, thereby resulting in different values of *ε_r_*. Under the same modifiers, the permittivity of nanocomposites at 60 vol % loading is larger than that at 40 vol % loading, as shown in Figure 6a. The immanent cause is the higher filler permittivity compared with pure PTFE. The increase in dielectric loss from loading results from a higher leakage current related to the aggregation of Ag/TiO_2_ fillers. For the nanocomposites at 40 vol % filler loading, the loss of nanocomposites that contain OPA-modified Ag/TiO_2_ NPs is higher than that obtained through modification by PFBPA. The trend of dielectric loss is similar to the nanocomposites at 60 vol % of Ag/TiO_2_ NPs. The possible reasons behind this observation are the superior adsorption of Ag/TiO_2_ NP by PFBPA over OPA, as supported by TGA, and the good compatibility of the fluorinated aryl group on the PFBPA modifier with the fluorinated polymer host [20].

**Figure 7:**
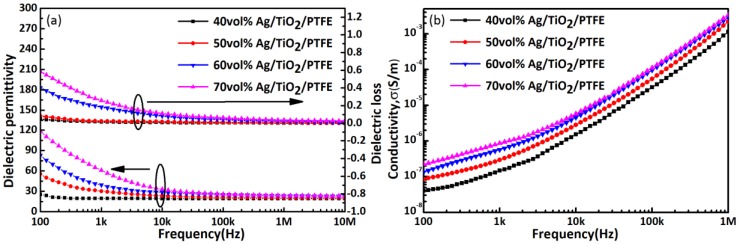
Frequency dependence of (**a**) dielectric performance and (**b**) conductivities σ for Ag/TiO_2_/PTFE nanocomposites containing PFBPA-modified Ag/TiO_2_ fillers at varying volume fractions at room temperature.

Figure 7a shows the frequency dependency of *ε_r_* and loss for Ag/TiO_2_/PTFE nanocomposites that contain various volume fractions of PFBPA-modified Ag/TiO_2_ fillers at 100 Hz–10 MHz at room temperature. The *ε_r_* at 100 Hz of nanocomposites with 70 vol % PFBPA-modified Ag/TiO_2_ NPs reaches ~120, which is almost sixty times higher than the pure PTFE matrix, and larger than that of most nanocomposites based on the Ag/TiO_2_ structure [13,15,23]. The underlying cause of this is the enlarged interfacial area through the accumulating charge at the interface between Ag, TiO_2_, and PTFE [29]. Hence, when Ag/TiO_2_ NPs uniformly disperse in the PTFE matrix, microcapacitors will form to obtain a higher *ε_r_*. Compared with the dielectric properties of rutile TiO_2_/PTFE without Ag cores, *ε_r_* of the rutile Ag/TiO_2_/PTFE nanocomposites greatly improves, because the modifiers maintain the homogeneity of inorganic particles in the organic matrix to further improve *ε_r_* [31]. At the same time, the maximal loss is suppressed to ~0.58, and remains stable and low at high frequencies (10 kHz–10 MHz), which may satisfy application requirements at high frequencies. Figure 7b shows the frequency dependence of conductivity for PFBPA-modified Ag/TiO_2_/PTFE nanocomposites with different volume fractions at room temperature. The conductivity of the nanocomposite increases linearly with increasing frequency. In general, conductivities σ of modified nanocomposites are all less than 5 × 10^–7^ S/m at 100 Hz at room temperature and at the test voltage of 500 mV, thereby corresponding to a low loss. The TiO_2_ shells suppress the formation of conducting paths through Ag NPs, thereby ensuring the insulating property of the nanocomposites.

**Figure 8:**
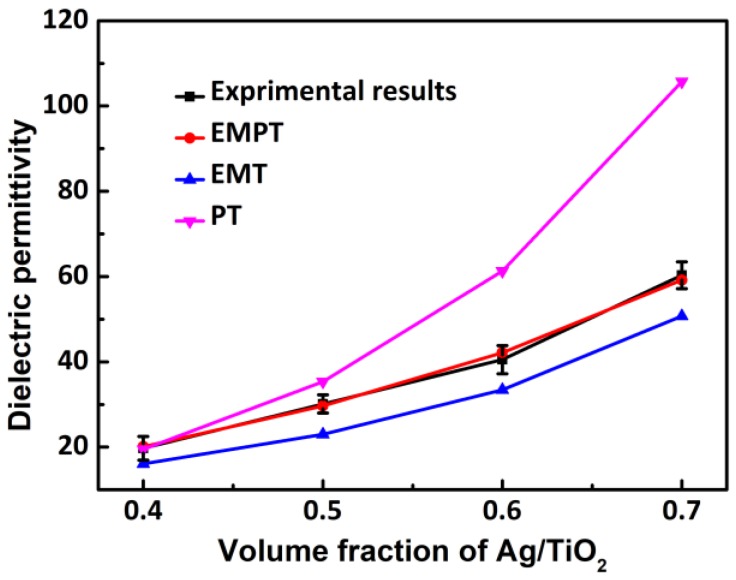
Experimental and theoretic values of *ε_r_* of Ag/TiO_2_/PTFE composites with different volume fractions of Ag/TiO_2_ at 1 kHz and at room temperature.

The *ε_r_* of nanocomposites can be related to the relevant constitutional parameters of composite materials, such as the *ε_r_* of the filler and the matrix, their interactions, filler shape, and connectivity [32]. Various models for predicting the dielectric permittivity of nanocomposites at 1 kHz and room temperature are as follows:

(1) $$\mathrm{PT}\;\mathrm{model}:\;\varepsilon =\varepsilon_{1}{\left|(f_{c}-f_{m})/f_{m}\right|}^{-q}$$

(2) $$\mathrm{EMT}\;\mathrm{model}:\;\varepsilon =\varepsilon_{1}[1+\frac{f_{cer}(\varepsilon_{2}-\varepsilon_{1})}{\varepsilon_{1}+n(1-f_{cer})(\varepsilon_{2}-\varepsilon_{1})}]$$

(3) $$\mathrm{EMPT}\;\mathrm{model}:\;\varepsilon =\varepsilon_{1}[1+\frac{f_{cer}(\varepsilon_{2}-\varepsilon_{1})}{\varepsilon_{1}+n(1-f_{cer})(\varepsilon_{2}-\varepsilon_{1})}]{\left|(f_{c}-f_{m})/f_{m}\right|}^{-q}$$

where *f_c_* is the percolation threshold of the metal filler; *ε*, *ε_1_*, and *ε_2_*, are the relative permittivities of the nanocomposites, polymer matrix, and ceramic, respectively; *f_cer_* and *f_m_* are the volume fractions of the ceramic and metallic filler, respectively; and *n* and *q* are the ceramic morphology fitting factor and the critical exponent, respectively. In the PT model (Equation (1)), both the ceramic (TiO_2_ shells) and polymer (PTFE) are regarded as the equivalent matrix, and the dielectric permittivity of the equivalent matrix changes with the volume fraction of Ag/TiO_2_ fillers. The deviation of experimental results from the PT model indicates that the Ag/TiO_2_ filler does not undergo the percolation mechanism. A possible reason for this is that the PT model empirically explains the percolation mechanism in polymeric nanocomposites with conductive fillers [33]. The EMT model (Equation (2)) predicts the *ε_r_* of ceramic/polymer composites, assuming that Ag/TiO_2_ is a hybrid filler [34]. The equivalent *ε_r_* of the Ag/TiO_2_ hybrids is approximately 10,000, which suggests that the high dielectric permittivity value of the nanocomposites is a result of the high equivalent *ε_r_* in the Ag/TiO_2_ hybrid filler. A similar explanation was reported by Rong Sun [28]. According to Figure 8, the experimental results are higher than the predicted values of the EMT model at a high volume fraction (>50 vol %) for the agglomeration of fillers and the destruction of the polymer continuity. The predicted value of the EMPT model agrees with the experimental results with *ε_1_* = 2.1, *ε_2_* = 110, *f_c_* = 0.31, *n* = 0.01, *q* = 0.4. In general, the percolation threshold varies from 0.004 to 0.24, depending on the aspect ratio and the nature of the conductive fillers [27,28,35]. The *f_c_* value of Ag/TiO_2_/PTFE is larger by 0.31 than the previously reported values, which possibly results from TiO_2_ shells cutting off the conductive path of the electronics in Ag, and the improved dispersion of the modified filler by phosphonic acids. The EMPT model (Equation (3)) is commonly applicable to triphasic metal/ceramic/polymer composites, thereby indicating that the dielectric mechanism of Ag/TiO_2_/PTFE nanocomposites is the same as that of a triphasic composite.

## 4. Conclusions

In summary, Ag/TiO_2_ core–shell NPs were fabricated, and polymer PTFE-based composites that contain modified Ag/TiO_2_ core–shell NPs were synthesized. FTIR and TGA showed that phosphonic acids form robust chemical bonds with TiO_2_ shells. Under the same filler volume fraction, the dielectric properties of PFPBA-modified Ag/TiO_2_/PTFE nanocomposites were relatively superior to those of OPA-modified nanocomposites. SEM showed that PFBPA-modified Ag/TiO_2_ fillers in the PTFE matrix were more uniformly dispersed than in OPA-modified fillers. Enhanced dielectric properties of the modified Ag/TiO_2_/PTFE nanocomposites were achieved. The permittivity at 100 Hz for PFPBA-modified Ag/TiO_2_/PTFE nanocomposites at room temperature was found to be ~120 and ~0.58. The large increase in the dielectric constant was mainly ascribed to the core-shell structure, while phosphonic acids suppressed the loss. Hence, phosphonic acids improve the dielectric property of core-shell Ag/TiO_2_/PTFE nanocomposites, and PFBPA forms a robust surface coverage of Ag/TiO_2_ NPs. Theoretical models on the nanocomposite predict that the EMPT model for metal/ceramic/polymer composites matches the experimental results best. The nanocomposites have potential applications in embedded devices and high-frequency fields in electronics.
